# Prenatal Diagnosis of Aorto-Left Ventricular Tunnel With Dysplastic Bicuspid Aortic Valve: From Fetal Cardiac Failure to Favorable Outcome

**DOI:** 10.3389/fped.2020.00069

**Published:** 2020-02-27

**Authors:** Ba Luu Truong, Anne Moreau De Bellaing, Emmanuelle Vialle, Ayman Haydar, Pascal Vouhe, Pierre Simon Jouk, Gerard Blaysat

**Affiliations:** ^1^Pediatric Cardiac Unit, Hôpital Universitaire de Grenoble-Alpes, Grenoble, France; ^2^Cardiovascular Unit, Children's Hospital 2, Ho Chi Minh City, Vietnam; ^3^Department of Pediatric Cardiac Surgery, Hôpital Universitaire Necker-Enfants Malades, APHP, Paris, France; ^4^Université Paris Descartes, Sorbonne Paris Cité, Paris, France; ^5^Cardiovascular Unit, Hôpital d'Annecy-Genevois, Annecy, France

**Keywords:** prenatal diagnosis, congenital heart defect, aorto-left ventricular tunnel, left ventricular function, aortic regurgitation

## Abstract

Aorto-left ventricular tunnel (ALVT) is a rare congenital heart defect. Surgery has to be performed early to avoid life-threatening complications. Prenatal diagnosis of this defect is challenging. We report a case of ALVT diagnosed in a fetus showing premature severe cardiac failure at 24 GA. The new born was operated at day 3 of life with good results. Two years later, he is still doing well recovering a complete normal cardiac function. ALVT should be suggested in front of any fetal cardiac failure. Thanks to early diagnosis, prompt neonatal management can be organized and allows positive outcome.

## Introduction

Aorto-left ventricular tunnel (ALVT) is an extremely rare defect (0.001 to 0.1% of all congenital heart defects with a male predominance) (1, 2). This abnormal paravalvular extracardiac communication between the ascending aorta and the left ventricle was first reported in an infant by Levy et al. (3).

Age of diagnosis and symptoms depend on the size of the ALVT, the severity of the aortic regurgitation caused by the defect, and the associated lesions. Overall, patients with ALVT are operated once the diagnosis is established given the risk of cardiac failure related with the shunt and the aortic insufficiency (4).

Fetal diagnosis of ALVT was first reported in 1995 in an autopsy study (1). Despite the recent improvement in routine ultrasound screening, prenatal diagnosis is still challenging and rare. Moreover, in the previous series, only a few newborns antenatally detected have survived whatever the performed postnatal cares (5–18).

Aortic origin of tunnel may be situated anywhere above the aortic sinus. The size and the shape of ALVT are variable (a large tubular, or saccular, protuberance on the anterior aspect of the aortic root). The tunnel ventricular orifice opens into the left ventricular cavity as in the majority of cases at the level of the fibrous triangle (90%) (4). While etiology of aorto-ventricular tunnel is unknown, the onset, severity, and progression of heart failure is, however, quite variable, and ranges from asymptomatic compensation to severe cardiac failure, sudden death, or even death in utero. This spectrum may reflect variable according to associated malformations (2).

We report the case of a fetus with ALVT and aortic dysplastic bicuspid valve early diagnosed during the pregnancy. Written informed consent for the publication was obtained from the parents.

## Case Report

**Figure 1:**
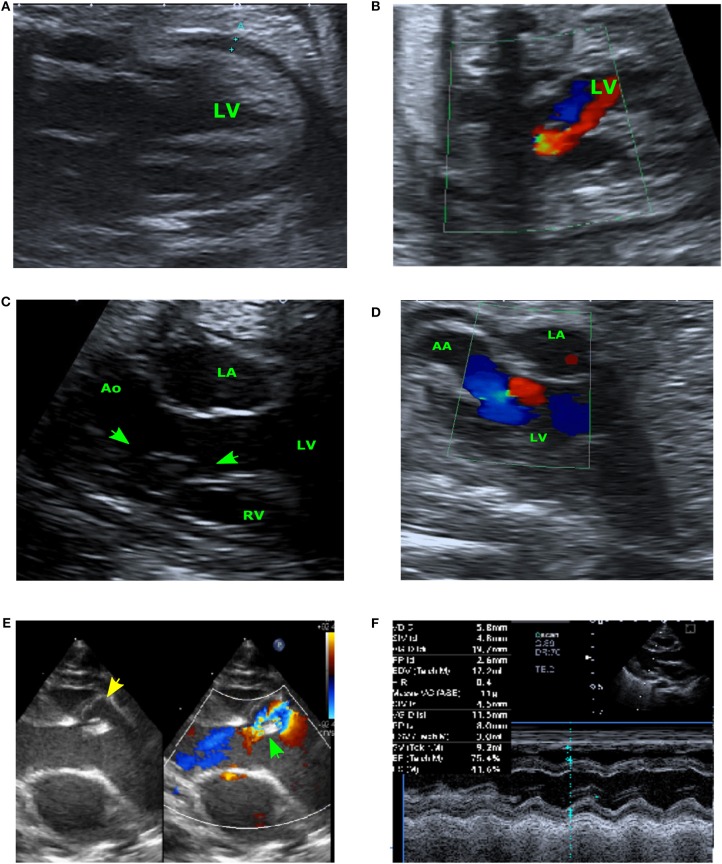
Prenatal echocardiogram at 24SG **(A,B)**. **(A)** Four-chambers view showing the cardiac effusion and the enlarged LV wall. **(B)** Aortic flow Doppler in early systole showing anterograde LV ejection (in blue) and important regurgitation caused by ALVT (in red) –Prenatal echocardiogram at 28 SG **(C,D)**. **(C)** Long-axis view showing the ALVT with its LV orifice (arrow) and its aortic orifice (star). **(D)** Modified aortic across view: Doppler flow of ALVT. Neonatal echocardiography **(E,F)**. **(E)** Pre-operative long-axis view showing the ALVT (the left-side image shows the yellow jet of the tunnel, and the right-side image shows the aliasing flow in the ventricular extremity of tunnel); the green arrow indicates the annular aortic valve). **(F)** Post-operative TM-view showing the restoration of the left ventricular function after surgery.

A fetus was referred at 24 gestational weeks (GW) for an abnormal aortic valve with severe regurgitation. We found a left ventricular (LV) dilatation caused by an important aortic regurgitant flow not through the aortic valve but through a tunnel near the interventricular septum. The left ventricular ejection fraction (LVEF) was altered (35 to 40%) and we also observed a hyper echogenic endocardial line of fibrosis. This cardiac failure led to a pericardial effusion (5 mm) and other manifestations of hydrops (ascites and moderate pleural effusion). According to all the sonographic findings, the diagnosis of ALVT was established (Figures 1A,B). Fetal karyotype was normal.

The repeated ultrasound examinations confirmed the diagnosis (Figures 1C,D) and showed a stabilization of the cardiac function over time. At 28 GW, the pericardial effusion was judged physiologic (<3 mm) and at 38 GW, the LVEF was still measured between 35 and 40%.

A male infant weighting 2,480 g and measuring 46 cm was delivered at term by caesarian section for maternal reason. Neonatal adaptation was good and the baby did not present signs of heart failure. Physical examination was normal (BPM 130, BP 82/42/55 mmHg) except of a loud systolodiastolic murmur. Chest X-ray established a heart/chest ratio at 0.68. Transfontanellar and abdominal ultrasound screenings were normal.

Transthoracic echocardiography confirmed the diagnostic of ALVT (Figure 1E) with mild aortic valvar regurgitation. The LV was notably dilated (20 mm) with moderate dysfunction.

Cardiac surgery was performed on day 3 of life. The aortic valve was found bicuspid including a postero-right leaflet, an antero-left leaflet, and a median raphe. The tunnel aortic orifice was localized above and left the right coronary ostium, close to the raphe. By median sternotomy under cardiopulmonary bypass, the ALVT was closed only on its aortic end using a heterologous pericardial patch avoiding distortion of the aortic cusps. The post-operative course was uneventful and the infant was discharged at day 8. The follow-up at 2 years demonstrated a positive outcome without any required treatment. There was no residual ALVT shunt but the aortic valve remained thick with mild regurgitation. The function of the left ventricle was completely normalized (Figure 1F).

## Discussion

ALVT anatomy in our case was consistent with most of those previously published. The tunnel aortic orifice was localized at the level of the right coronary Valsalva sinus, above the sinotubular junction, as 80% of cases (4). Then, the ALVT coursed inferiorly and leftward, anterior to the aortic annulus, posterior to the pulmonary trunk and the sub-pulmonary muscular conus, and entered the LV superior to the ventricular septum, immediately below the aortic valve under the commissure separating the right and left coronary sigmoid (2). Prenatal diagnosis of ALVT is challenging. Just a few cases, including autopsy series, were reported in the literature. ALVT diagnosis should be considered in front of all unexplained fetal cardiac failures associated with a significant aortic regurgitant flow and an aortic root dilatation (5–8, 8–10, 12–14, 17–20). The massive regurgitation might be confused with aortic valvar insufficiency. In other cases, the decrease of the ratio Pulmonary Artery/Aorta initially suggested the diagnosis of tetralogy of Fallot (2, 5, 6, 12–14, 17). Specific ALVT ultrasound findings were described such as the “cockade sign” but not established in our case (14). Diagnosis is definitive when echocardiography demonstrates an abnormal tunnel-like structure connecting the ascending aorta with the left ventricle.

ALVT can cause severe aortic regurgitation and be responsible for early congestive heart failure in infants. Post-operative and long-term outcome are good encouraging rapid surgery once diagnosis is established. Prenatal diagnosis is helpful for organizing premature neonatal management. This avoids turbulence-related damages to the leaflets or progressive aneurysmal dilation of the aortic root as seen in patients surgically repaired at an older age (21).

ALVT are associated with other cardiac defects in around 45% of cases (22–26). Most of them imply coronary arteries or outflow tract valves. As aortic valvar anomalies are reported (bicuspid and unicuspid valve) and can cause a residual aortic regurgitant flow after ALVT management, a long-term follow-up is essential (27–29).

Most of the prenatal diagnosed cases are very serious and characterized by severe diastolic cardiac dysfunction and poor outcome compared with those diagnosed postnatally. Improvement of the cardiac function in our case might suggest a relative reduction of tunnel diameter during the fetal period. That could explain the favorable outcome of some rare cases. This should be known when counseling parents during pregnancy.

## Conclusion

ALVT should be considered in any fetus with aortic regurgitation and/or cardiac failure especially when associated with pulmonary/aorta ratio decrease. Early diagnosis allows prompt neonatal management. After adequate surgical repair whatever the closure technique, left ventricular function might improve even in case of severe premature fetal cardiac failure.

## Data Availability Statement

The datasets generated for this study are available on request to the corresponding author.

## Ethics Statement

The studies involving human participants were reviewed and approved by the Ethics Committee of Centre Hospito-Universitaire de Grenoble. The patients/participants provided their written informed consent to participate in this study.

## Author Contributions

BT, PJ, and GB conceived the presented idea and realized echos. AM and AH realized echos and helped with intensive care. EV followed the patient. PV was the cardiac surgeon. All authors discussed the results and contributed to the final manuscript.

### Conflict of Interest

The authors declare that the research was conducted in the absence of any commercial or financial relationships that could be construed as a potential conflict of interest.
